# Reusable Fe_2_O_3_/TiO_2_/PVC Photocatalysts for the Removal of Methylene Blue in the Presence of Simulated Solar Radiation

**DOI:** 10.3390/nano13030460

**Published:** 2023-01-23

**Authors:** Ivana Jagodić, Imre Guth, Svetlana Lukić-Petrović, Dragana Tamindžija, Daniela Šojić Merkulov, Nina Finčur, Szabolcs Bognár, Predrag Putnik, Nemanja Banić

**Affiliations:** 1 Department of Chemistry, Biochemistry and Environmental Protection, Faculty of Sciences, University of Novi Sad, Trg Dositeja Obradovića 3, 21000 Novi Sad, Serbia; 2 Department of Physics, Faculty of Sciences, University of Novi Sad, Trg Dositeja Obradovića 4, 21000 Novi Sad, Serbia; 3 Department of Biology and Ecology, Faculty of Sciences, University of Novi Sad, Trg Dositeja Obradovića 2, 21000 Novi Sad, Serbia; 4 Department of Food Technology, University North, Trg dr. Žarka Dolinara 1, 48000 Koprivnica, Croatia

**Keywords:** methylene blue, Fe_2_O_3_/TiO_2_/PVC composites, photodegradation, photo-cleaning, photo-Fenton process, photocatalyst reusing

## Abstract

Currently, environmental pollution by various organic pollutants (e.g., organic dyes) is a serious, emerging global issue. The aqueous environment is highly exposed to the harmful effects of these organic compounds. Furthermore, the commonly applied conventional purification techniques are not sufficient enough. Heterogeneous photocatalysis and the photo-Fenton process are effective, low-cost and green alternatives for the removal of organic pollutants. In this study, different iron(III) oxide/titanium(IV) oxide/polyvinyl chloride (Fe_2_O_3_/TiO_2_/PVC) nanocomposites in tablet form were investigated in the photodegradation of methylene blue (MB) under simulated sunlight, and their possible antibacterial effects were examined. The newly synthesized nanocomposites were characterized by scanning electron microscope, X-ray diffraction, UV–Vis diffuse reflectance spectroscopy, and Raman spectroscopy. The results showed a *hematite* crystal form in the case of Fe_2_O_3(2)_ and Fe_2_O_3_ samples, while the Fe_2_O_3(1)_ sample showed a combination of *hematite* and synthetic mineral *akaganeite*. The highest photocatalytic efficiency was achieved in the presence of Fe_2_O_3_/TiO_2_/PVC, when 70.6% of MB was removed. In addition, the possible photo-cleaning and reuse of the mentioned photocatalyst was also examined. Based on the results, it can be seen that the activity did not decrease after five successive runs. Nanocomposites also exhibited mild antibacterial effects against the two tested Gram-positive bacteria (*S. aureus* and *B. cereus*).

## 1. Introduction

Many companies use dyes to color their products, which uses a lot of water. Consequently, a sizable volume of effluent is created [1]. These aesthetically unappealing water-soluble paints also consume dissolved oxygen and reduce the amount of radiation that passes through the water’s surface. Based on the aforementioned impacts, these pollutants harm aquatic life and have major negative consequences on the environment that have an impact on human health [2]. This is because they are toxic and have cancer-causing properties.

The thiazine color family includes the heterocyclic aromatic chemical methylene blue (MB). It is dark blue when it is oxidized. It is frequently utilized in numerous photoreactions, in the creation of photosensors, and for medicinal applications due to its antifungal impact because of its exceptionally sensitive and uncommon features. It is a common industrial dye that causes major health issues such as elevated heart rate, vomiting, diarrhea, and jaundice at greater doses. Therefore, it is crucial to remove this contaminant from wastewater [3,4].

Unfortunately, due to their photocatalytic stability and resistance to chemical oxidation, the traditional biochemical and physicochemical water purification technologies are unsuitable for the removal of textile dyes. These dangerous dyes have been eliminated from aqueous environments using a variety of methods, including chemical precipitation, sedimentation, adsorption onto large surface carriers [5], biological membranes, and ion exchange processes. These techniques, however, have not shown to be as effective because to their usual slowness, expensive equipment requirements, incomplete elimination of contaminants, and requirement of additional procedures and energy for the complete removal of the chemicals in question [6]. In order to breakdown the organic dyes more effectively while using less energy, new removal techniques should be devised [7].

Advanced oxidation technologies (AOPs), which convert pollutants into less damaging byproducts, offer promising substitutes for the removal of colors. Since photocatalytic degradation is a sustainable, affordable, and ecologically beneficial technology, it is receiving a lot of attention these days. The limited usage of semiconductors as photocatalysts, for instance, limits the practical application of this process and is one of the disadvantages of this technology. The development of a photocatalyst with high photocatalytic efficiency, recyclability, and high activity in sunlight should be prioritized [8]. In order to compare the behavior and effectiveness of different studied photodegradation processes, MB has frequently been used as a model compound for photocatalytic degradation. In addition, this azo dye comparatively slows the photodegradation process, making it resistant to UV exposure and easier to analyze. These are the main reasons why methylene blue was used in this study [9].

The photocatalytic process, which results in an electron transfer from the occupied valence band to the vacant conductor band, is often started by UV radiation interacting with semiconductors. On the photocatalyst surface, negative electron (e^−^) and positive hole (h^+^) pairs will consequently develop. The e^−^–h^+^ pairs are either engaged in redox processes, mineralizing the organic contaminants, or are recombined, resulting in decreased photocatalytic activity. Mineralization results from reactions between the target pollutant and, for instance, peroxide and superoxide radicals, which are created when a positive hole and electrons react with water and oxygen [10,11,12,13]. Nano-TiO_2_ has been regarded as one of the most suitable catalysts in the degradation of many harmful organic pollutants [6] due to its stable chemical structure, biocompatibility, great oxidizing power, non-toxicity, and low cost [14,15].

Fast e^−^–h^+^ pair recombination and the activity being almost entirely restricted to the UV range are two significant drawbacks of inorganic photocatalysts. In order to boost future applications, much work has been put toward moving their absorption wavelengths to the visible region. To enhance charge separation and increase the catalyst’s lifespan during mineralization, transition metal elements such as Fe are frequently included [16,17,18,19]. The photocatalytic activity of titanium has improved due to characteristics such as the small band gap energy of Fe^3+^ (1.9 eV), half-filled electronic configuration, and the similarity of the ionic radius of Fe^3+^ (0.64 Å) with the coordinated Ti^4+^ (0.68 Å).

In addition to heterogeneous photocatalysis, the traditional homogeneous Fenton method, which generates highly reactive hydroxyl radicals (^•^OH) by activating H_2_O_2_ with Fe^2+^, is another AOP that has been effectively used [20,21]. The Fenton process has some limitations that prevent it from being used in many situations, including: (i) a limited pH working range (pH < 3), (ii) high iron concentrations in final effluents that request for costly removal before discharging processes, and (iii) a high iron content in the sludge that takes a significant amount of H_2_O_2_ [22]. Due to a heterogeneous Fenton process’ great efficiency, high pH values, and durability, these shortcomings have been overcome [23].

It was established from previous studies using ESR analysis and different kind of scavengers that ^•^O_2_^−^ and ^•^OH radicals are the main responsible species for the degradation of MB. The created e^−^ and h^+^ are transferred to the photocatalyst surface. The e^−^ reduces O_2_ to superoxide radical anions (^•^O_2_^−^), while the h^+^ either oxidizes H_2_O to ^•^OH or directly oxidizes MB dye. These reactive species (^•^O_2_^−^, ^•^OH and h^+^) initiate the redox reactions and degrade MB dye into CO_2_, H_2_O, and inorganic ions. Thus, the MB dye solution becomes colorless due to the degradation of aromatic rings. Additionally, it was shown that the importance of the participation of certain species decreases in the following order: (e^−^) > (^•^O_2_^−^) > (^•^OH) > (h^+^) in the photocatalysis mechanism of composites under SSR [24,25].

The contribution of this study is the synthesis of several TiO_2_-based catalysts immobilized on PVC in the form of nanocomposite tablets. Additionally, a straightforward composite preparation that only requires a few steps and does not include the use of more expensive materials was realized. The removal of MB from aqueous solutions using newly synthesized nanocomposites (Fe_2_O_3_/PVC, Fe_2_O_3_/TiO_2_/PVC, Fe_2_O_3(1)_/PVC, Fe_2_O_3(1)_/TiO_2_/PVC, Fe_2_O_3(2)_/PVC, and Fe_2_O_3(2)_/TiO_2(1)_/PVC) was also studied. These composites’ photocatalytic effectiveness were examined with and without simulated solar radiation (SSR). To more precisely assess the photodegradation effectiveness of the newly created TiO_2_-based nanocomposite, a reusability test was also conducted.

## 2. Materials and Methods

### 2.1. Chemicals and Solutions

All chemicals were of reagent grade and used without purification. MB (C_16_H_18_ClN_3_S, >97%) was manufactured by Kemika (Zagreb, Croatia); H_2_SO_4_ (95–97%) was provided from Merck (Darmstadt, Germany); NaOH (ZorkaPharm, Šabac, Serbia); Fe_2_O_3_ (commercial nanopowder, grain sizes <50 nm, Merck, Germany), Fe_2_O_3(1)_ (synthesized hematite nanoparticles by [26], Fe_2_O_3(2)_ (synthesized hematite nanoparticles by [27]). Commercial TiO_2(1)_ (Hombikat, CAS No 13463-67-7, surface area 35–65 m^2^/g and 21 nm primary particle size, anatase, Sigma-Aldrich Chemie GmbH, Steinheim, Germany) and TiO_2_ (Molar Chemicals KFT, Halásztelek, Hungary) were also used for the synthesis of the nanocomposite. All solutions were prepared using ultrapure water. The aqueous stock solution of MB had a concentration of 2.45 × 10^−2^ mM.

### 2.2. Photocatalyst Preparation

For preparing Fe_2_O_3_/(TiO_2_)/PVC composites, a commercial patented formulation of PVC and differently synthesized catalysts were used. In all Fe_2_O_3_/TiO_2_ materials, the Fe content in relation to TiO_2_ was 7.2% *w/w*, and the percentage of composites in the final sample with PVC was 2.5%. In our previous study, six materials using PVC were created, with the following composite percentages: 1.0%, 1.75%, 2.5%, 3.75%, 5.0%, and 7.5%. According to the results of the measurements, the removal of MB is best accomplished with a composite content of 2.5% on PVC [25]. As a result, 2.5% of the composite was employed in all experiments. The mixture was first made by physically mixing a few drops of ultrapure water with equal mass ratios of Fe_2_O_3_/TiO_2_ (2.5%) catalyst in PVC. The mixture was then drawn using a hand-held dough-making machine with stainless steel rollers into thin sheets that were 2 mm thick. Afterward, a round cutter with a 5 mm diameter was used to create composite tablets. The obtained tablets were put in a laboratory beaker filled with water and boiled for 15 min. After being thoroughly cleaned with ultrapure water, the tablets were solidified for 30 min at 140 °C in an oven. Using this procedure, six composites with different contents of nanocatalysts: Fe_2_O_3_/PVC, Fe_2_O_3_/TiO_2_/PVC, Fe_2_O_3(1)_/PVC, Fe_2_O_3(1)_/TiO_2_/PVC, Fe_2_O_3(2)_/PVC, and Fe_2_O_3(2)_/TiO_2(1)_/PVC were prepared. The same procedure was used to prepare the PVC composite without adding nanocatalysts.

### 2.3. Characterization

Using a JSM-6460LV JIEL microscope, scanning electron microscopy (SEM) images were obtained. The X-ray diffraction (XRD) was performed with a Rigaku MiniFlex 600 goniometer, using Cu K_α1,2_ (secondary graphite monochromator) radiation at 15 mA and 40 kV, and a step scan mode of 0.03° s^−1^, 2 s per step in a 2*θ* range from 3° to 80°, which allowed for successful profile fitting with PDXL and HighScore Plus (PANanalytical, Malvern, UK) software (v3.0). The Centic MMS Raman spectrometer, which utilizes a charge-coupled device as a detector, was used to measure the samples. As the excitation source, a 70 mW diode laser operating at 785 nm (1.58 eV) was applied. Using an Ocean Optics QE65000 High-Sensitivity Fiber Optic Spectrometer (Dunedin, FL, USA), the diffuse reflectance spectra were measured. Spectra Suite Ocean Optical software was then used to estimate the Kubelka–Munk function. Each measurement was performed at room temperature. Traditional sample preparation methods—grinding with MgO or KBr powder and pressing—could not be used for the two optical measurements. In order to use them for measurement, solid-prepared samples were recorded on their flat surfaces. While the optical spectra obtained in this way do not alter in character, they cannot be taken as absolute measurements. The samples’ reflection spectra were used to determine the band characteristics because they are already opaque in the visible spectrum region.

### 2.4. Removal Activity Test

**Figure 1:**
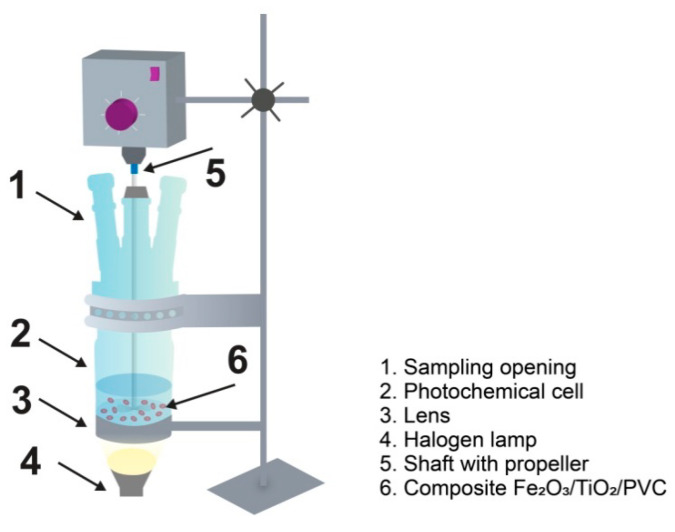
Schematic diagram of the photocatalytic reactor [25]. Reproduced with permission from [25]. Copyright Springer, 2022.

A batch reactor composed of Pyrex glass (Figure 1) was used for the experiments (total volume of ca. 170 mL, solution depth of 65 mm). In the presence of SSR (*I*_UV_ = 0.223 mW/cm^2^; *I*_Vis_ = 208.5 mW/cm^2^), the potential removal of 30 mL of MB solution using nanocomposites was examined. The halogen lamp was used as an SSR source (Philips, Amsterdam, The Netherlands; type: MR16/50W/GU10/240V). Under the lens, the halogen lamp was positioned. To 30 mL of aqueous solution, 29 tablets of Fe_2_O_3_/TiO_2_/PVC composite were added. In our previous study, it was found that up to 10% of the MB was photodegraded when the number of utilized tablets was 3, 7, and 14. The highest amount of MB was photodegraded utilizing 29 tablets, with a photodegradation efficiency of 21.4% and a 57.7% in overall removal efficiency. The efficiency of photodegradation decreases as the quantity of tablets increases further. This is the reason for using 29 tablets in the experiments [25]. After that, the photoreactor was mounted on the lens so that the radiation could be focused at the suspension. An overhead stirrer was used to mix the reactor’s contents continually. The stirrer shaft had a diameter of 6 mm, and the propeller blades were 10 × 7 mm in size. Three fans were used to cool the reactor, which had a 44 °C temperature.

### 2.5. Photocatalyst Reuse and Photo-Cleaning

**Figure 2:**
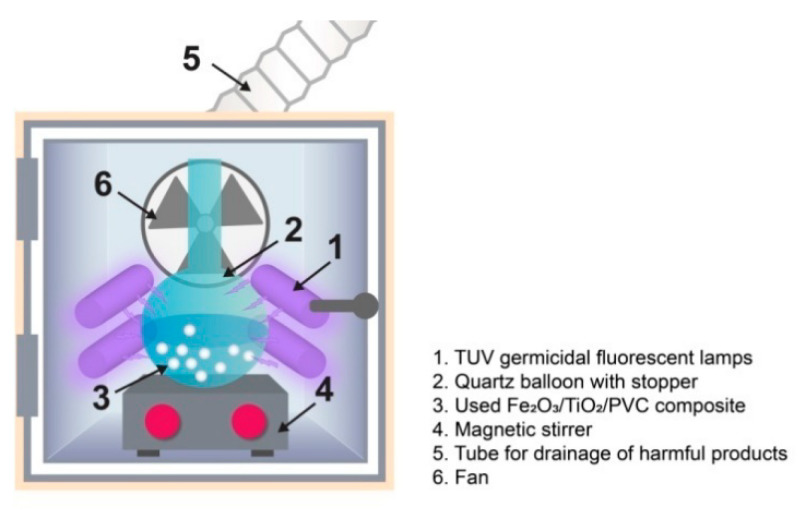
Schematic diagram of the laboratory-made photo-cleaning chamber [25]. Reproduced with permission from [25]. Copyright Springer, 2022.

A magnetic stirring bar, 30 mL of ultrapure water, and 29 tablets of Fe_2_O_3_/TiO_2_/PVC photocatalyst were placed inside a quartz balloon. After that, the quartz balloon was sealed and placed on a magnetic stirrer in a photo-cleaning chamber constructed in the lab (Figure 2), and the mixture was exposed to UVC radiation for 60 min. The UVC source consisted of a group of four 6 W TUV germicidal fluorescent lamps (*λ*_max_ = 253.7 nm, Philips, The Netherlands, type: TUV 6 W). One pair of lamps was placed on either side of the quartz flask, sideways to it. The UVC radiation applied had an intensity of *I*_UVC_ = 3.025 mW/cm^2^. The hazardous UVC radiation byproducts were removed from the air by a fan.

Using spectrophotometry, the photo-cleaning of the 29 used Fe_2_O_3_/TiO_2_/PVC photocatalyst tablets and the possibility of their reuse were analyzed. After 60 min of tablet irradiation, the solution’s absorption spectra were recorded from 200 to 800 nm.

### 2.6. Antibacterial Activity Testing of Solid Samples

The agar diffusion method was used for testing the antibacterial activity of photocatalysts and was performed according to the standard EUCAST method [28] with a modification of incubation temperature. An incubation temperature of 22 °C was used instead of 37 °C to imitate the real conditions of catalyst application more closely. Briefly, after 24 h of incubation, bacterial culture was used to prepare 0.5 MacFarland standard suspensions of bacterial strains, corresponding to a bacterial count of 1 × 10^8^ cells/mL. The suspension was spread across the surface of Mueller Hinton agar plates and left to dry for 5–10 min. Then, photocatalyst tablets were placed on the surface of the plate alongside an antibiotic disk containing gentamicin, which was used as a control. Plates were then incubated for 18 ± 2 h when inhibition zones around the disks were measured in millimeters. Four different bacteria were tested: two Gram-positive bacteria (*Staphylococcus aureus* ATCC 25923 and *Bacillus cereus* ATCC 14579) and two Gram-negative bacteria (*Escherichia coli* ATCC 25922 and *Pseudomonas aeruginosa* ATCC 35554). These bacteria were chosen, as they are commonly used for testing of antibacterial properties of plastic and other materials.

### 2.7. Antibacterial Activity Testing of Treated Water Solutions and Cleaning Solutions

The antibacterial activity of treated water solutions and cleaning solutions was determined by the microdilution method and *Pseudomonas putida* growth inhibition test.

The microdilution method was performed according to the standard CLSI method [29] with modification of incubation temperature as mentioned previously. Briefly, after 24 h of incubation, bacterial culture was used to prepare 0.5 MacFarland standard suspension of bacterial strains, corresponding to a bacterial count of 1 × 10^8^ cells/mL. The suspension was diluted 100 times and inoculated to microplate wells containing geometrical dilutions of samples (final tested sample concentrations were 50, 25, 12.5, 6.25, 3.125, 1.56, 0.78%). Plates were incubated for 18 ± 2 h, after which the growth of bacteria was monitored by reading absorbance at 600 nm using a Multiskan GO (Thermo Scientific, Waltham, MA, USA) microplate reader. The lowest sample concentration without detectable bacterial growth was recorded as minimal inhibitory concentration (MIC). The lowest concentration that killed at least 99.9% of bacteria was recorded as minimal bactericidal concentration (MBC).

The *Pseudomonas putida* growth inhibition test was performed according to the standard method ISO 10712 [30]. Briefly, *Pseudomonas putida* culture was set to the absorbance of 0.065 at 600 nm in a preculture medium and incubated for 5 h at 150 rpm and 23 °C. Then, the suspension was diluted to an absorbance of 0.15 and mixed with an equal amount of test medium. This mixture was mixed with liquid samples, resulting in a final concentration of 80% of the sample. A standard solution of dichlorophenol was used as a control. Growth control contained saline solution instead of samples. Microplates were incubated 16 h at 23 °C. Bacterial growth was measured by the Multiskan GO (Thermo Scientific, USA) microplate reader at the start and end of incubation. The percentage of growth inhibition (*I* (%)) was determined based on starting and ending absorbance of samples and growth control.

### 2.8. Presence of Fungal Contamination in Treated Water Solutions and Cleaning Solutions

Fungal contamination in liquid samples was detected by spread plating 100 µL of samples onto Malt extract agar and incubating it for 7–14 days at 26 °C. If fungal growth appeared, slides were prepared and observed by bright field microscopy on Olympus BX-51 to identify the fungi.

## 3. Results and Discussion

### 3.1. Characterization of the Synthesized Photocatalyst

#### 3.1.1. X-ray Diffraction Analysis of Fe_2_O_3_/(TiO_2_)/PVC Photocatalysts

XRD patterns of pure PVC, Fe_2_O_3_, and TiO_2_ from different manufacturers and modified Fe_2_O_3_/TiO_2_/PVC samples are presented. XRD spectrum of PVC (Figure S1) exhibits clear Bragg diffraction peaks that confirm the predominantly crystalline character of the sample. Our previous research also found a somewhat unexpected result for the polymer [25]. The technological procedure of polymer synthesis should be held responsible for the observed considerable changes in polymer crystallinity with variations in molar mass and sample preparation.

Figure S2 shows the XRD spectra of Fe_2_O_3_. The XRD pattern of samples labeled Fe_2_O_3(2)_-III and Fe_2_O_3_-I exhibits the hexagonal crystal form of Fe_2_O_3_—*hematite*, with lattice parameters *a*= 0.5038 nm and *c*= 1.3772 nm (JCPDS Card No. 24-72). On the other hand, the sample labeled Fe_2_O_3(1)_-II is a combination of *hematite* and synthetic mineral *akaganeite*, i.e., a tetragonal form of iron oxide hydroxide, *β*-Fe(OH) (*a* = 1.0535 nm, *c* = 0.303 nm; JCPDS Card No. 34-1266) or orthorhombic form of iron oxide hydrate, *β*-Fe_2_O_3_∙H_2_O (*a* = 1.026 nm, *b* = 1.058 nm, *c* = 0.304 nm; JCPDS Card No. 08-0093).

Figure S3 shows the XRD spectra of TiO_2_. Obtained data based on the diffractogram indicate that the sample marked TiO_2(1)_-III is a mineral *anatase* (JCPDS Card No. 21-1272), while the sample marked TiO_2_-II is a combination of two minerals TiO_2_: *anatase* and *rutile* (JCPDS Card No. 21-1276).

Fe_2_O_3_/TiO_2_-doped PVC sample diffraction patterns are essentially identical to those of pure PVC (Figure S4). Namely, in all obtained spectra, the diffraction maxima of the crystalline PVC dominate, and only in traces can the presence of TiO_2_ in anatase form be observed (peaks at 2*θ* = 25.07; 47.50; 63.14 deg). This only shows that the concentration of titanium and iron oxides is below the detection threshold of the instrument.

#### 3.1.2. SEM Imaging of Fe_2_O_3_/(TiO_2_)/PVC Photocatalysts

**Figure 3:**
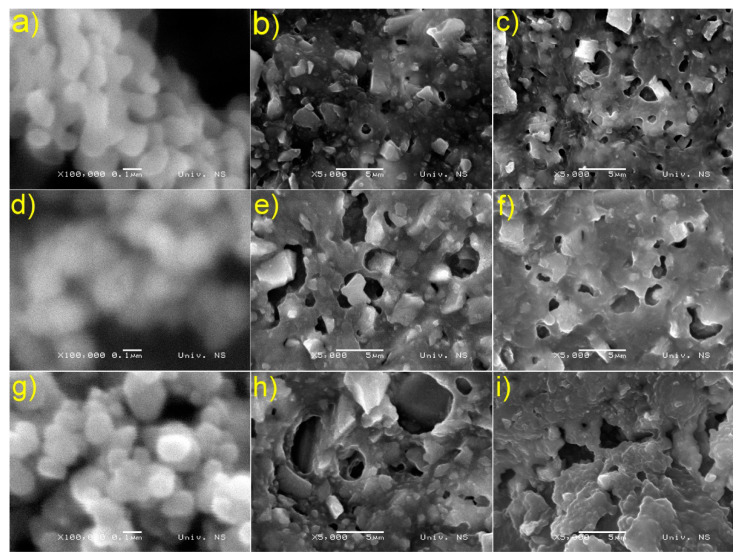
SEM images for: (**a**) unsupported Fe_2_O_3_, (**b**) Fe_2_O_3_/PVC,(**c**) Fe_2_O_3_/TiO_2_/PVC, (**d**) unsupported Fe_2_O_3(1)_, (**e**) Fe_2_O_3(1)_/PVC, (**f**) Fe_2_O_3(1)_/TiO_2_/PVC, (**g**) unsupported Fe_2_O_3(2)_, (**h**) Fe_2_O_3(2)_/PVC and (**i**) Fe_2_O_3(2)_/TiO_2(1)_/PVC.

The difference in the morphology of the unsupported Fe_2_O_3_ and synthesized composites is quite apparent and is shown in Figure 3. A distinct structure is seen in the case of unmodified Fe_2_O_3_ particles (Figure 3a,d,g), and their sizes range from 50 to 180 nm. On the contrary, the differences between synthesized materials are almost unobservable (Figure 3b,c,e,f,h,i). The generation of holes with sizes ranging from 0.94 to 6.1 μm is visible in all synthetic materials. Furthermore, it was found that the presence of TiO_2_ in the most efficient Fe_2_O_3_/TiO_2_/PVC material causes the holes to increase in size in contrast to the other composites, where the presence of TiO_2_ in the Fe_2_O_3_/PVC causes the holes to shrink.

#### 3.1.3. Raman Analysis of Fe_2_O_3_/(TiO_2_)/PVC Photocatalysts

Figures S5–S7 show recorded Raman spectra of components (PVC, Fe_2_O_3_ and TiO_2_) and prepared samples. For such a low impurity concentration, only the most intense peak of TiO_2_ (at the wavenumber around 634 cm^−1^) is noticeable on the obtained samples in addition to the structural spectrum of PVC [25,31]. Other peaks of TiO_2_ as well as Fe_2_O_3_ peaks are incorporated in the spectrum of PVC. In the spectra of pure Fe_2_O_3_ components, a wider band at wavenumbers 660–690 cm^−1^ is observed on the sample Fe_2_O_3(1)_/TiO_2_/PVC, which does not exist in the other samples. This band most likely belongs to the variant of *akaganeite*, which is in accordance with the results obtained in XRD measurements (Figure S2).

#### 3.1.4. UV–Vis DRS of Fe_2_O_3_/TiO_2_/PVC Photocatalysts

The reflection spectra of the samples, together with the spectrum of PVC, are shown in Figure S8. On these spectra, a significant shift in the reflection spectra toward longer wavelengths is observed, i.e., significant changes in the energy gap in the energy diagram due to the presence of impurities (addition) in PVC. The difference in the reflection spectrum of the mixtures from PVC shows the absorption of electromagnetic radiation in samples, which can be attributed to the impurities in PVC. For all three samples, this absorbance is similar and can be deconvoluted (a Gaussian line profile is taken) into three bands (in Figure S9 marked with B1, B2, and B3). The solid lines in the figure are the absorbances based on the measured value, while the dashed lines are the results of calculations (convolution of the three bands). The same figure also shows the bands obtained by deconvolution on the Fe_2_O_3(1)_/TiO_2_/PVC sample (curves B1, B2, and B3). These absorption bands in all three samples are in the visible area, almost at the same place (Table 1), with little variation in their intensity and width.

Using the Kubelka–Munk function *F*(*R*) *=* (1*−R*)^2^/2*R*, the optical band gap was calculated from observed diffuse reflectance (*R*) spectra of prepared samples [32]. Optical bandgap energies were obtained as the energy of onset at the low-energy side of the plot (*F*(*R*)*hν*)*^2^* from photon energy (Figure S10) for all bands separately. These obtained energy values are found in Table 2 and show the position of the additional levels located in the energy gap of the carrier (PVC). The positions of these levels are quite similar (the values in brackets are determined with a large error due to the shape of the reflection curves). In the magnified graphic of the PVC in Figure S10, it is clearly seen that Band B2 belongs to the PVC itself. In the same picture, in the case of the Fe_2_O_3_/TiO_2_/PVC sample, another smaller band is visible below band B1, somewhere at 3.15 eV.

### 3.2. Results of Photocatalytic Experiments on MB Degradation

#### 3.2.1. Study of MB Removal Efficiency Using Fe_2_O_3_/TiO_2_/PVC Photocatalysts

**Figure 4:**
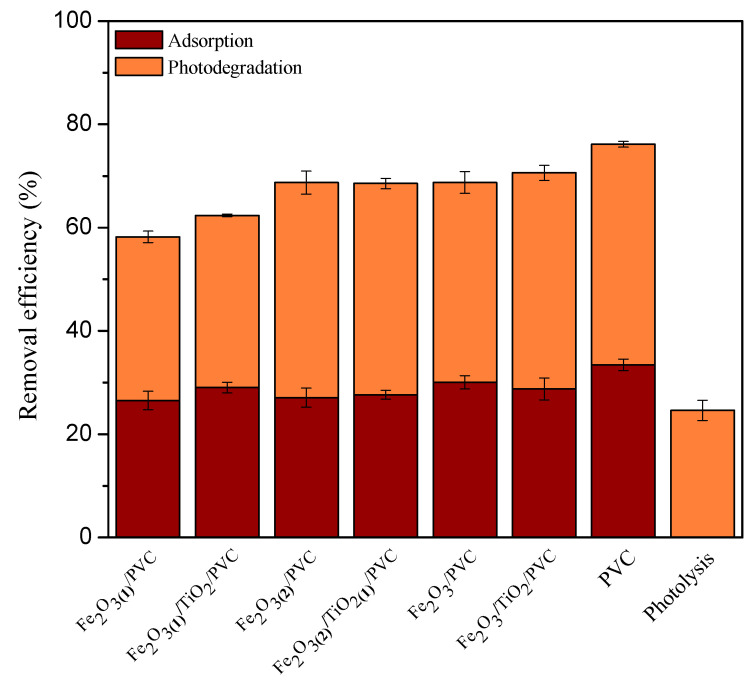
Contribution of adsorption and photodegradation under SSR (calculated after 60 min of processes) for MB removal efficiency (*c*_0_ = 2.45∙10^−2^ mM) in the presence of 29 tablets of Fe_2_O_3_/TiO_2_/PVC nanocomposites at a stirring rate of 490 rpm.

**Figure 5:**
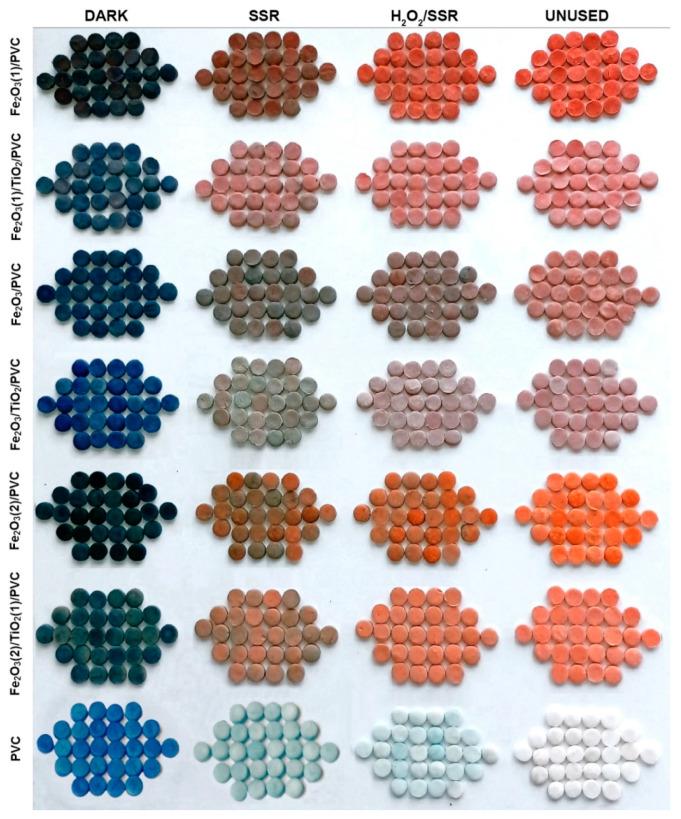
Photos of tablets after 180 min of the MB removal (*c*_0_ = 2.45∙10^−2^ mM) at a stirring rate of 490 rpm: first column (adsorption in the dark), second column (removal in the presence of SSR), third column (removal in the presence of SSR and H_2_O_2_) and fourth column (unused tablets).

Prior to examining the photodegradation efficiency on the overall MB removal, the adsorption rate was determined for each composite, in dark (Figure 4 and Figure S11). As can be shown, all synthetic materials demonstrated a considerable MB adsorption efficiency after 60 min, ranging from 26.5% for Fe_2_O_3(1)_/PVC composites to 33.4% for PVC supports. The increased adsorption level could be explained by two factors. Firstly, as the metal oxide content increased, the increasing surface area of the adsorbent provided more binding sites for MB [33]. Furthermore, the metal oxidized surface was negatively charged at higher pH [34], which facilitates interaction with cationic MB. Besides the adsorption, the efficiency of photocatalytic degradation under SSR was also examined in the removal of MB (Figure 4 and Figure S11). Comparing the removal efficiency after 60 min of irradiation to the same period of adsorption in the dark, there was a noticeable improvement. Namely, the following MB removal percentages were achieved: 42.7% for PVC support, 38.7% for Fe_2_O_3_/PVC, 41.8% for Fe_2_O_3_/TiO_2_/PVC, 31.7% for Fe_2_O_3(1)_/PVC, 33.3% for Fe_2_O_3(1)_/TiO_2_/PVC, 41.7% for Fe_2_O_3(2)_/PVC, and 40.9% for Fe_2_O_3(2)_/TiO_2(1)_/PVC. The acquired data indicate that the presence of SSR has a beneficial influence on the overall effectiveness of MB removal for all composites under consideration, with PVC supports and Fe_2_O_3_/TiO_2_/PVC composites exhibiting the highest activity. The overall removal efficiency of MB, considering both the adsorption and photodegradation, was 76.2% for PVC, 68.8% for Fe_2_O_3_/PVC, 70.6% for Fe_2_O_3_/TiO_2_/PVC, 58.2% for Fe_2_O_3(1)_/PVC, 62.4% for Fe_2_O_3(1)_/TiO_2_/PVC, 68.7% for Fe_2_O_3(2)_/PVC, and 68.6% for Fe_2_O_3(2)_/TiO_2(1)_/PVC. The presence of SSR is also noticeable in Figure 5 which shows the appearance of the tablets after 180 min of removal. The irradiated tablets had lighter color than the non-irradiated ones, indicating that along with adsorption, the photodegradation process also occurred in the presence of radiation. Figure 4 shows that after 60 min of irradiation, the overall MB removal efficiency in the presence of SSR for all studied composites was higher than direct photolysis (24.61%). It is also observed that the contribution of photodegradation in the presence of SSR for all studied composites was higher than the efficiency of direct photolysis. According to the thorough systematics of the results, for all composites under study, the contribution of photodegradation to the overall effectiveness of MB removal was much higher than the contribution of the adsorption process.

#### 3.2.2. Effect of H_2_O_2_ Concentration and pH on MB Degradation Efficiency

**Figure 6:**
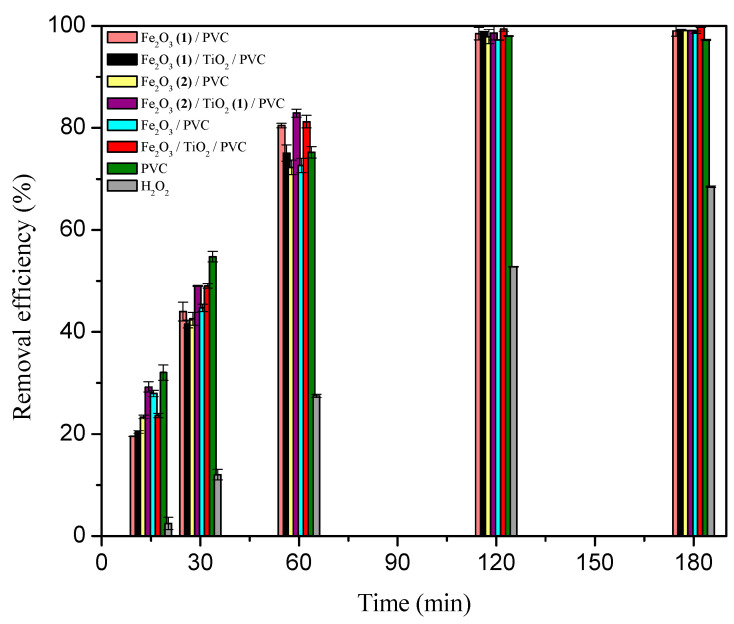
MB removal efficiency (*c*_0_ = 2.45∙10^−2^ mM) in the presence of 29 tablets of Fe_2_O_3_/TiO_2_/PVC composites, 13.1 mM H_2_O_2_, at a stirring rate of 490 rpm under SSR.

Hydrogen peroxide can increase the pollutant removal efficiency by creating additional hydroxyl radicals in the presence of Fe_2_O_3_ (Fenton process) [35]. The effect of H_2_O_2_ concentration on the efficiency of MB degradation was studied in the range of 6.5–196.5 mM. Figure 6 illustrates that the Fe_2_O_3_/TiO_2_/PVC composite had the highest MB removal effectiveness (99.7%) after 180 min of irradiation in the presence of 13.1 mM hydrogen peroxide. On the other hand, the lowest MB removal efficiency was obtained for the PVC composite (97.2%) and H_2_O_2_ without the presence of photocatalysts (68.5%). Based on the presented results, the Fe_2_O_3_/TiO_2_/PVC composite was chosen for the study of the influence of H_2_O_2_ concentration on the MB removal efficiency.

**Figure 7:**
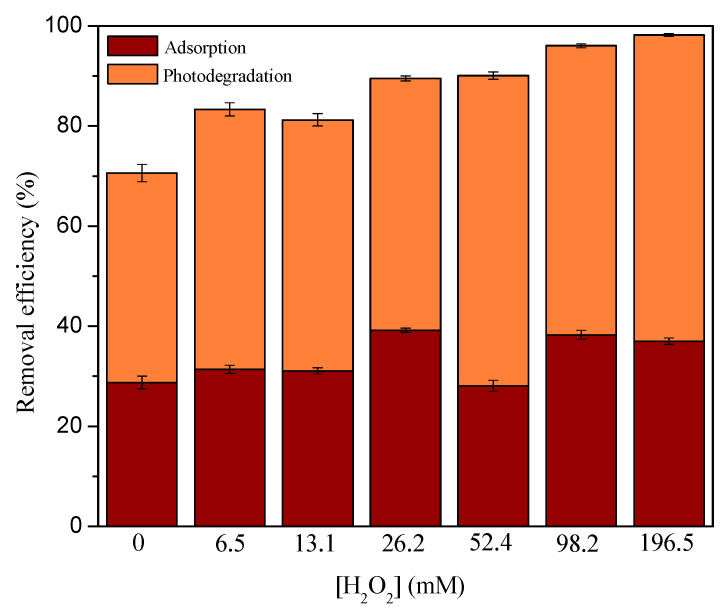
Contribution of adsorption and photodegradation under SSR (calculated after 60 min of processes) for MB removal efficiency (*c*_0_ = 2.45∙10^−2^ mM) in the presence of 29 tablets of Fe_2_O_3_/TiO_2_/PVC composites and different H_2_O_2_ concentrations at a stirring rate of 490 rpm.

For each hydrogen peroxide concentration, adsorption effectiveness in the dark was determined (Figure 7 and Figure S12). Figure 7 illustrates the considerable MB removal effectiveness of the Fe_2_O_3_/TiO_2_/PVC composite by adsorption in the presence or absence of H_2_O_2_, with the lowest value without H_2_O_2_ (28.8%) and the greatest value using 26.2 mM H_2_O_2_ (39.1%). Figure 7 and Figure S12 suggest that increasing the concentration of H_2_O_2_ to 196.5 mM also increases the MB removal efficiency (98.2%), while the efficiency decreases slightly at higher doses (above 98.2 mM). The first increase in H_2_O_2_ concentration enhanced the degradation efficiency due to the effect of the produced ^•^OH radicals. However, at higher doses, H_2_O_2_ acts as a potential ^•^OH scavenger. Therefore, adding H_2_O_2_ above its optimal concentration can lead to the formation of hydroperoxyl radicals (^•^H_2_O_2_), which are much less reactive and do not contribute to the oxidative degradation of organic compounds [36].

**Figure 8:**
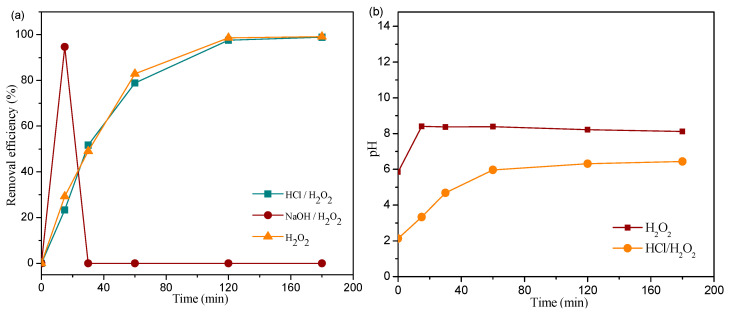
Influence of the initial value of pH solution on the removal efficiency of MB (c_0_ = 2.45∙10^−2^ mM) in the presence of 29 tablets of Fe_2_O_3(2)_/TiO_2(1)_/PVC composites and 13.1 mM H_2_O_2_ at a stirring rate of 490 rpm: (**a**) kinetic curve and (**b**) change in pH value during removal process under SSR.

The initial pH value was investigated regarding the effectiveness of removing MB in the presence of Fe_2_O_3(2)_/TiO_2(1)_/PVC composites and 13.1 mM H_2_O_2_. Figure 8a indicates the modest differences in MB removal efficiency between the system with no pH adjustment (pH 5.8) compared to the one with HCl present, where the initial pH was 2.1. The initial pH of the solution after the addition of NaOH was 11.8, and at this pH, when MB is in its colorless leucomorphic form, it is unable to observe the removal of MB at 660 nm [37]. The pH of the solution was monitored while the MB was being removed. Despite the divergence of the initial pH value, after 60 min of treatment, the pH stabilized and did not change: the pH value for the hydrogen peroxide/hydrochloric acid system was 6.0, and the pH value for the sodium hydroxide/hydrogen peroxide system was 8.3. (Figure 8b).

#### 3.2.3. MB Removal with the Reused Fe_2_O_3_/TiO_2_/PVC Photocatalyst

**Figure 9:**
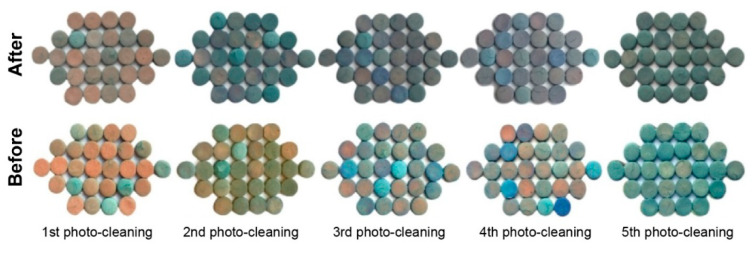
The 29 tablets of Fe_2_O_3_/TiO_2_/PVC composites after each photo-cleaning step used in the MB (*c*_0_ = 2.45∙10^−2^ mM) removal under the influence of UVC irradiation for 60 min.

Technology for water treatment depends on the ability to regenerate a photocatalyst so that it can keep its photocatalytic activity without altering its original chemical composition [38]. For the purpose of investigating the viability of photo-cleaning and reuse, the Fe_2_O_3_/TiO_2_/PVC/SSR system was selected. Figure 9 shows that the composites nearly totally regained their original appearance following the initial photo-cleaning, losing the blue color impurities that resulted from the adsorbed MB. The absorption spectrum of ultrapure water remaining after the photo-cleaning of composites (Figure S13), which shows that after the first photo-cleaning, the least organic matter, and after the fifth time, the most organic matter, remained in ultrapure water, further supports the effectiveness of photo-cleaning. This tendency might be explained by the degradation of more adsorbed MB and/or the decomposition of the composites themselves as a result of repeated use.

This photo-cleaning method’s distinctive advantage is underlined by the fact that it consumes less energy (germicidal UVC fluorescent lamps) while also causing residual organic matter in utilized water to be decomposed during photo-cleaning tablets. On the other hand, PVC would degrade if the temperature were increased, a method frequently employed for catalyst regeneration [27]. Another benefit of this method is that no additional chemicals are employed, and the purification is carried out in a sustainable manner.

**Figure 10:**
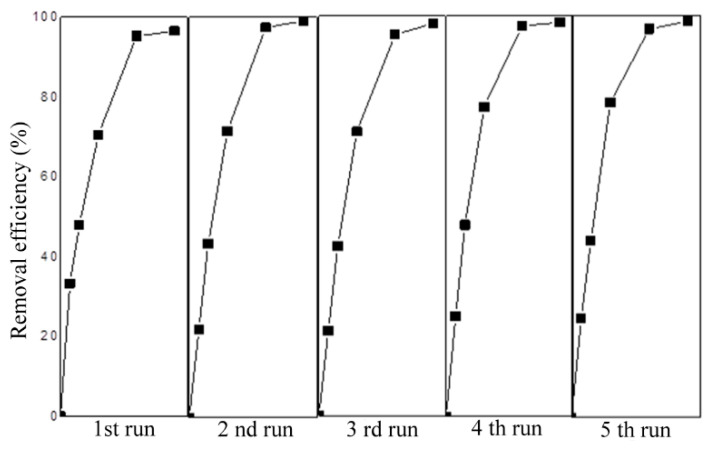
Kinetic curves for the reuse of 29 tablets of Fe_2_O_3_/TiO_2_/PVC composites in the case of MB (*c*_0_ = 2.45∙10^−2^ mM) removal under SSR, at a stirring rate of 490 rpm.

In addition to the effectiveness of photo-cleaning composites, the potential for their reuse was investigated. It is evident that each reuse caused MB to decompose into different intermediates from the absorption spectra of MB at the beginning and after removal with purified Fe_2_O_3_/TiO_2_/PVC composites in the presence of SSR (Figure S14). Additionally, Figure 10 shows that even after five consecutive applications, the removal effectiveness of MB using Fe_2_O_3_/TiO_2_/PVC composites remains unchanged.

### 3.3. Antibacterial Activity of Nanocomposites, Treated Water Solutions and Cleaning Solutions

The antibacterial activity determined by the agar diffusion method showed that Gram-negative bacteria were insensitive to tested composites, whereas a mild inhibitory effect was observed against Gram-positive bacteria (Table 3, Figure S15). Specifically, *B. cereus* was inhibited by PVC and Fe_2_O_3_/PVC composites, while all three composite samples inhibited *S. aureus*. An antibiotic disk with gentamicin was used as a control and gave expected diameters of inhibition.

Antibacterial activity of treated water solutions and cleaning solutions was also tested. Microdilution assay revealed that *E. coli*, *P. aeruginosa* and *B. cereus* were not inhibited by any of the samples, while the growth of *S. aureus* was inhibited by dye solution of MB and dye solution after photolysis (samples 1 and 2) (Table 4). The concentration of 12.5% of sample 1 and 25% of sample 2 was enough to inhibit the further growth of *S. aureus*. MBC was not detected for samples 1 and 2, indicating that they only stop further multiplication of bacterial cells (they have an inhibitory effect on bacteria), but do not kill already present bacterial cells (they do not have a bactericidal effect).

*Pseudomonas putida* growth inhibition test showed that liquid samples in a concentration of 80% exert mild growth inhibition in the range of 10.68 to 23.03%. For samples 1 and 2, standard methods could not be applied because of their intensive coloration.

Fungal contamination of liquid samples was noted after prolonged storage of samples. Namely, *Fusarium* sp. was detected in samples 2 and 3, while *Penicillium* sp. was noted in sample 4. Other samples had no fungal contamination. These fungi are common aerocontaminants of various samples and have probably contaminated samples during the experimental procedures and processing.

## 4. Conclusions

In this research, the efficiency of six newly synthesized nanocomposite tablets in the photocatalytic removal of MB was investigated. Furthermore, their possible antibacterial effect was examined.

XRD analysis showed a hexagonal *hematite* crystal form in the case of Fe_2_O_3(2)_ and Fe_2_O_3_ samples, while the Fe_2_O_3(1)_ sample is a combination of *hematite* and synthetic mineral *akaganeite*. The Raman spectroscopy measurements also proved the mentioned form, since a wider band at wavenumbers 660–690 cm^−1^ is observed on the sample Fe_2_O_3(1)_/TiO_2_/PVC, which most likely belongs to the variant of *akaganite*.

Based on the obtained photocatalytic experiments, it can be concluded that all newly synthesized composites had higher MB degradation efficiency compared to direct photolysis after 60 min of SSR. The highest activity was observed in the case of the Fe_2_O_3_/TiO_2_/PVC composite. Our findings also showed that the degradation efficiency of the investigated composites was improved in the presence of H_2_O_2_, due to the photo-Fenton process, while the initial pH did not have a significant effect on the photocatalytic activity. The possible photo-cleaning process and reuse of Fe_2_O_3_/TiO_2_/PVC tablets were also examined. The obtained results showed that the photocatalytic activity of the tablets did not decrease even after the fifth successive run.

Furthermore, the antibacterial studies determined that PVC, Fe_2_O_3_/PVC and Fe_2_O_3_/TiO_2_/PVC inhibited *S. aureus*, while PVC and Fe_2_O_3_/PVC composites inhibited *B. cereus*.

Our findings showed that Fe_2_O_3_/TiO_2_/PVC can be an appropriate candidate for the eco-friendly treatment of wastewater. Namely, heterogeneous photocatalysis harvests sunlight, which is a free and renewable source of energy, and the high activity of the mentioned composite under simulated sunlight additionally reduces the operation costs, since there is no need for an artificial irradiation source. Furthermore, the tablet form and high reusability of Fe_2_O_3_/TiO_2_/PVC also add up to the advantages of these nanocomposites, due to the easier separation from the aqueous environment, which makes the whole treatment process more accessible. On the other hand, further experiments should be carried out regarding the possible degradation mechanism pathways. By doing this, we could obtain a detailed image about the degradation intermediates of MB and could additionally improve the photocatalytic efficiency of the mentioned nanocomposite in order to reach complete mineralization. Even though the synthesis does not require harmful chemicals, expensive materials and use of high temperatures, various eco-inspired, plant-based synthesis techniques should also be developed in order to reduce our ecological footprint in nature.

## Figures and Tables

**Table 1:** Experimental data of absorption bands for samplesFe_2_O_3_/TiO_2_/PVC (FWHM—full width at half maximum, shown in nm).

Sample	B1			B2			B3		
	*λ*_max_ (nm)	FWHM	I	*λ*_max_ (nm)	FWHM	I	*λ*_max_ (nm)	FWHM	I
Fe_2_O_3_/TiO_2_/PVC	448	90	8.98	537	158	14.1	657	203	13.9
Fe_2_O_3(1)_/TiO_2_/PVC	450	128	26.5	544	129	37.7	690	314	22.1
Fe_2_O_3(2)_/TiO_2(1)_/PVC	443	67	19.4	533	88	29.6	651	77	6.01

**Table 2:** Energy positions of additional levels in Fe_2_O_3_/TiO_2_/PVC samples within the forbidden zone of PVC.

Sample	*E*_g_ (eV)		
	B1	B2	B3
Fe_2_O_3_/TiO_2_/PVC	3.22 3.15	(~2.6)	2.08
Fe_2_O_3(1)_/TiO_2_/PVC	3.26	(~2.6)	2.12
Fe_2_O_3(2)_/TiO_2(1)_/PVC	(~3.3)	2.72	2.16

**Table 3:** Antibacterial activity of photocatalysts determined by agar diffusion method.

Sample	Zone of Inhibition Diameter (mm)			
	*E. coli*	*P. aeruginosa*	*B. cer* *eus*	*S. aur* *eus*
Fe_2_O_3_/TiO_2_/PVC	0	0	0	6
PVC	0	0	6	8
Fe_2_O_3_/PVC	0	0	6	8
Gentamicin (control)	19	17	22	20

**Table 4:** The antibacterial activity of treated water solutions and cleaning solutions determined as minimal inhibitory concentration (MIC) by microdilution method and inhibition percentage *I* (%) by *Pseudomonas putida* growth inhibition test.

Sample No.	Sample Description	MIC (%)*				*I* (%) **
		*E. coli*	*P. aeruginosa*	*B. cer* *eus*	*S. aur* *eus*	
**1**	**Dye solution of MB**	No inhibition	No inhibition	No inhibition	12.5	Nd ^***^
2	Dye solution after photolysis				25	Nd ^***^
3	PVC/SSR				No inhibition	18.77
4	1st removal Fe_2_O_3_/TiO_2_/PVC/SSR					17.07
5	2nd removal Fe_2_O_3_/TiO_2_/PVC/SSR					10.68
6	3rd removal Fe_2_O_3_/TiO_2_/PVC/SSR					12.38
7	4th removal Fe_2_O_3_/TiO_2_/PVC/SSR					23.03
8	5th removal Fe_2_O_3_/TiO_2_/PVC/SSR					18.28
9	1st run cleaning					17.48
10	2nd run cleaning					20.26
11	3rd run cleaning					17.62
12	4th run cleaning					15.98
13	5th run cleaning					15.22

* Minimal inhibitory concentration determined by microdilution method for Escherichia coli ATCC 25922, Pseudomonas aeruginosa ATCC 35554, Bacillus cereus ATCC 14579 and Staphylococcus aureus ATCC 25923; ** growth inhibition in the presence of 80% of the sample determined by *Pseudomonas putida* growth inhibition test; *** not determined.

## Data Availability

Not applicable.

## Supplementary Materials

The following supporting information can be downloaded at: https://www.mdpi.com/article/10.3390/nano13030460/s1, Figure S1: XRD pattern of PVC samples; Figure S2: XRD pattern of Fe_2_O_3_; Figure S3: XRD pattern of TiO_2_; Figure S4: XRD pattern of Fe_2_O_3_/TiO_2_/PVC; Figure S5: Raman spectra of Fe_2_O_3_/TiO_2_/PVC at room temperature; Figure S6: Raman spectra of Fe_2_O_3(1)_/TiO_2_/PVC at room temperature; Figure S7: Raman spectra of Fe_2_O_3(2)_/TiO_2(1)_/PVC at room temperature; Figure S8: Reflection spectra of Fe_2_O_3_/TiO_2_/PVC; Figure S9: Absorption of Fe_2_O_3_/TiO_2_/PVC (solid lines—measured values; dashed lines—calculated convolutions, B1–B3 deconvoluated bands for Fe_2_O_3(1)_/TiO_2_/PVC sample); Figure S10: Energy plot of Fe_2_O_3_/TiO_2_/PVC samples; Figure S11:Adsorption and photodegradation kinetic curves for MB removal efficiency (*c*_0_ = 2.45∙10^−2^ mM) in the presence of 29 tablets of Fe_2_O_3_/TiO_2_/PVC nanocomposites at a stirring rate of 490 rpm; Figure S12: Adsorption and photodegradation kinetic curves for MB removal efficiency (c_0_ = 2.45∙10^−2^ mM) in the presence of 29 tablets of Fe_2_O_3_/TiO_2_/PVC composites and different H_2_O_2_ concentrations at a stirring rate of 490 rpm; Figure S13:Absorption spectra of ultrapure water left after each photo-cleaning of 29 tablets of Fe_2_O_3_/TiO_2_/PVC composites used in the MB (*c*_0_ = 2.45∙10^−2^ mM) removal under the influence of UVC irradiation for 60 min; Figure S14: Absorption spectra of MB (*c*_0_ = 2.45∙10^−2^ mM) at the beginning and after 180 min of SSR irradiation, at a stirring rate of 490 rpm for five consecutive removals; Figure S15: Antibacterial activity of photocatalysts (1—Fe_2_O_3_/TiO_2_/PVC, 2—PVC, 3—Fe_2_O_3_/PVC, 4—gentamicin (control)) determined by agar diffusion method: A —*Esherichia coli*, B—*Pseudomonas aeruginosa*, C—*Bacillus cereus*, D—*Staphylococcus aureus*.
