# Supplementary material for: Reusable Fe2O3/TiO2/PVC Photocatalysts for the Removal of Methylene Blue in the Presence of Simulated Solar Radiation
Source: Nanomaterials (Basel). 2023 Jan 23;13(3):460. doi: 10.3390/nano13030460 (PMC9919515; doi:10.3390/nano13030460)
Supplement: Supplementary file 1 [file nanomaterials-13-00460-s001.zip › nanomaterials-2160813-supplementary.pdf]

Supplementary Material

Reusable Fe_2O_3/TiO_2/PVC Photocatalysts for the Removal of Methylene Blue in the Presence of Simulated Solar Radiation

Ivana Jagodić [1], Imre Guth [2], Svetlana Lukić-Petrović [2], Dragana Tamindžija [3], Daniela Šojić Merkulov [1], Nina Finčur [1], Szabolcs Bognár [1], Predrag Putnik [4,*] and Nemanja Banić [1,*]

1 Department of Chemistry, Biochemistry and Environmental Protection, University of Novi Sad Faculty of Sciences, Trg Dositeja Obradovića 3, 21000 Novi Sad, Serbia
2 Department of Physics, University of Novi Sad Faculty of Sciences, Trg Dositeja Obradovića 4, 21000 Novi Sad, Serbia
3 Department of Biology and Ecology, University of Novi Sad Faculty of Sciences, Trg Dositeja Obradovića 2, 21000 Novi Sad, Serbia
4 Department of Food Technology, University North, Trg Dr. Žarka Dolinara 1, 48000 Koprivnica, Croatia
* Correspondence: pputnik@alumni.uconn.edu (P.P.); nemanja.banic@dh.uns.ac.rs (N.B.)

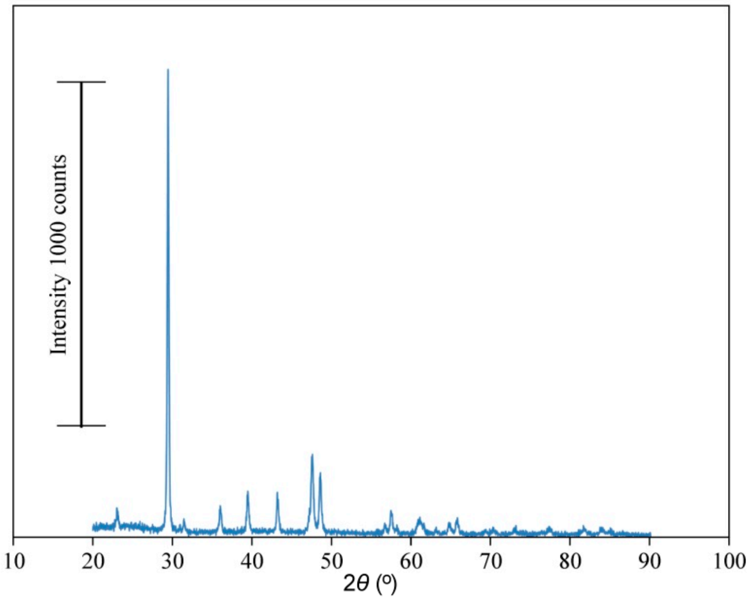

Figure S1. XRD pattern of PVC samples.

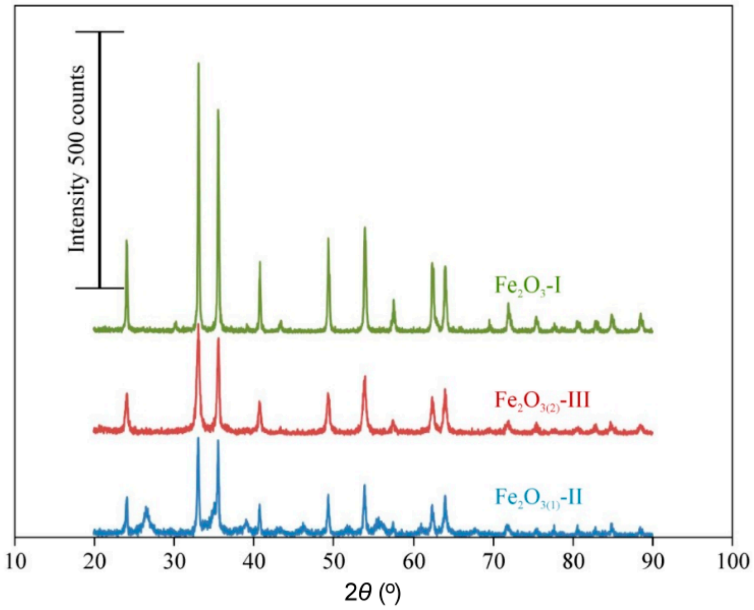

Figure S2. XRD pattern of Fe_2O_3.

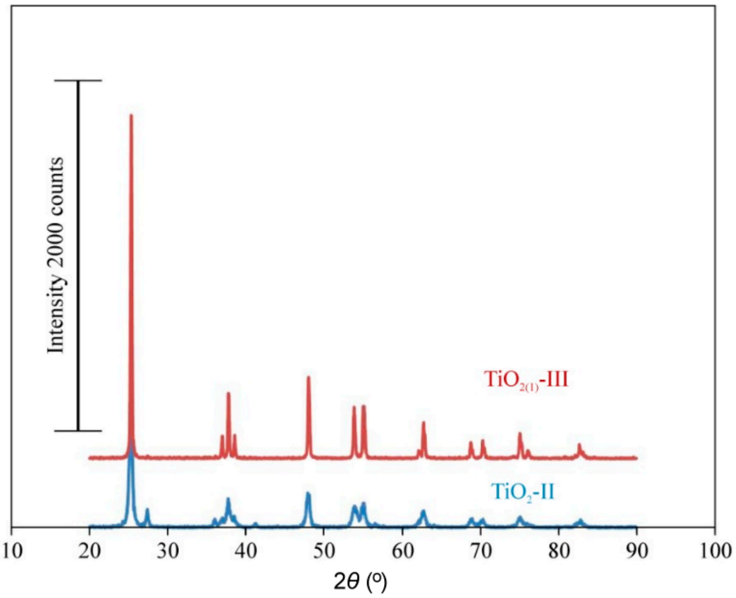

Figure S3. XRD pattern of TiO_2.

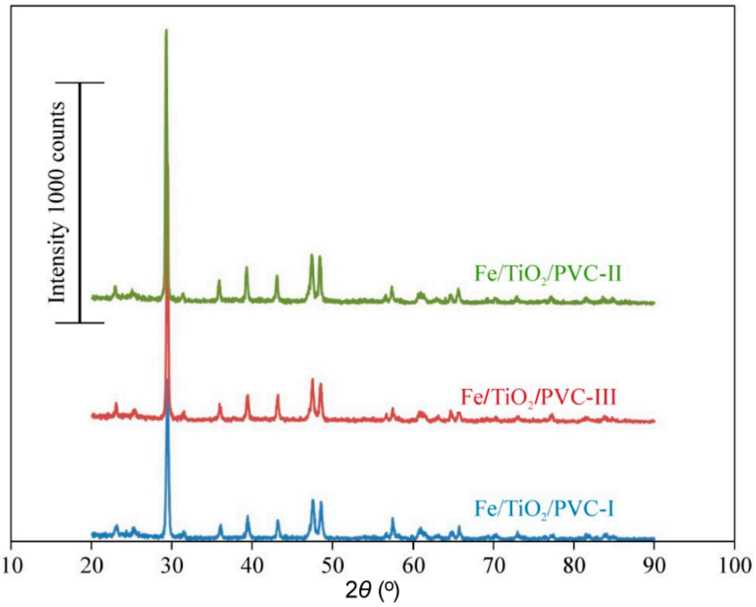

Figure S4. XRD pattern of $Fe_2O_3/TiO_2/PVC$.

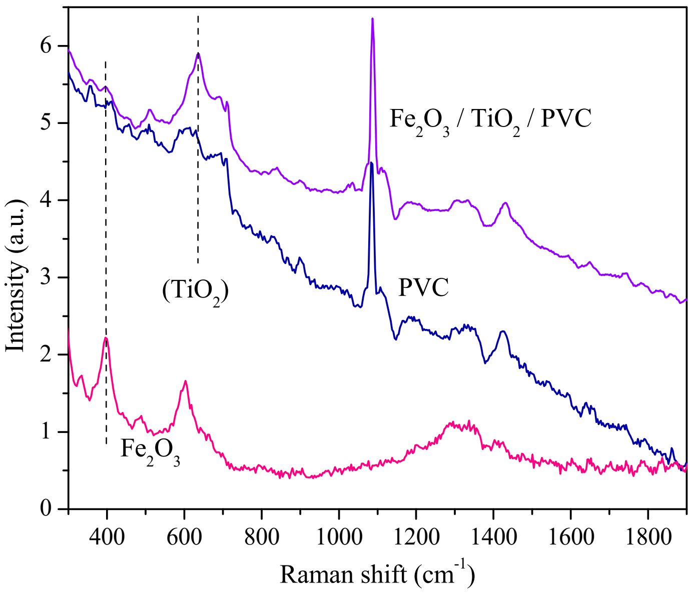

Figure S5. Raman spectra of Fe_2O_3/TiO_2/PVC at room temperature.

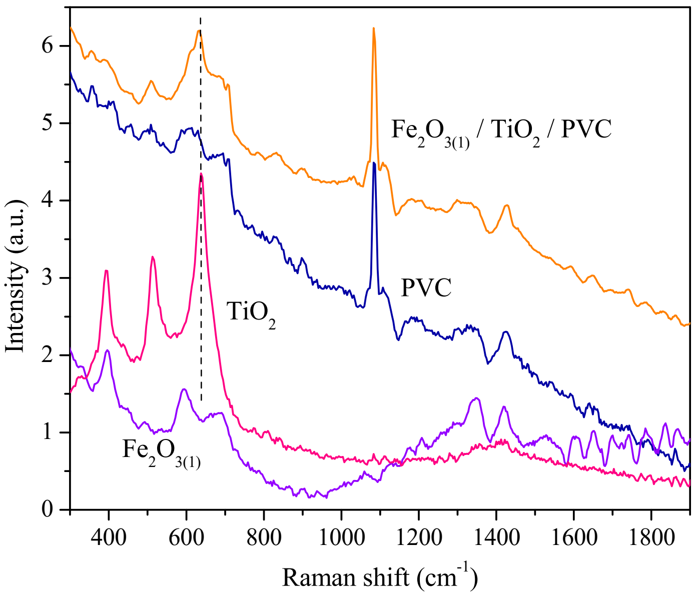

Figure S6. Raman spectra of $Fe_2O_{3(1)}/TiO_2$/PVC at room temperature.

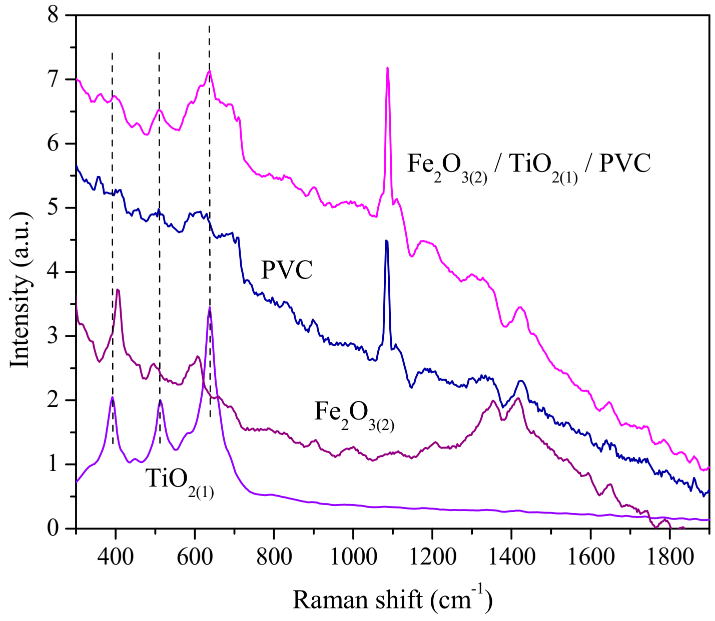

Figure S7. Raman spectra of $Fe_2O_{3(2)}$/$TiO_{2(1)}$/PVC at room temperature.

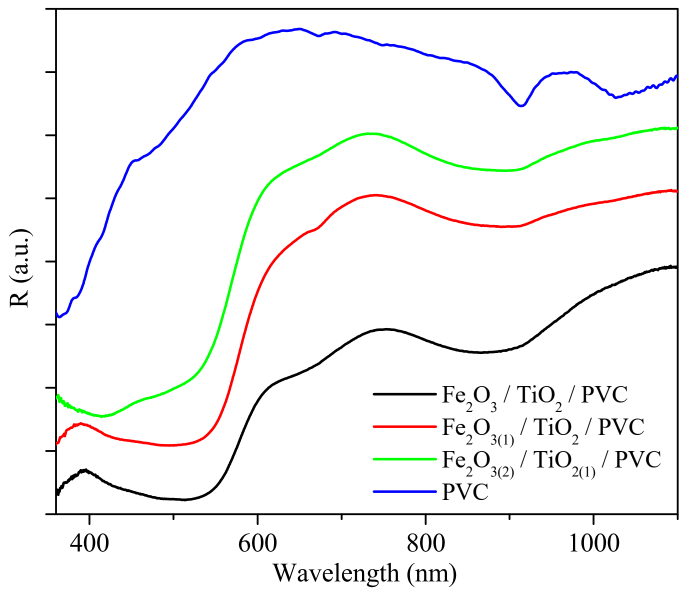

Figure S8. Reflection spectra of Fe_2O_3/TiO_2/PVC.

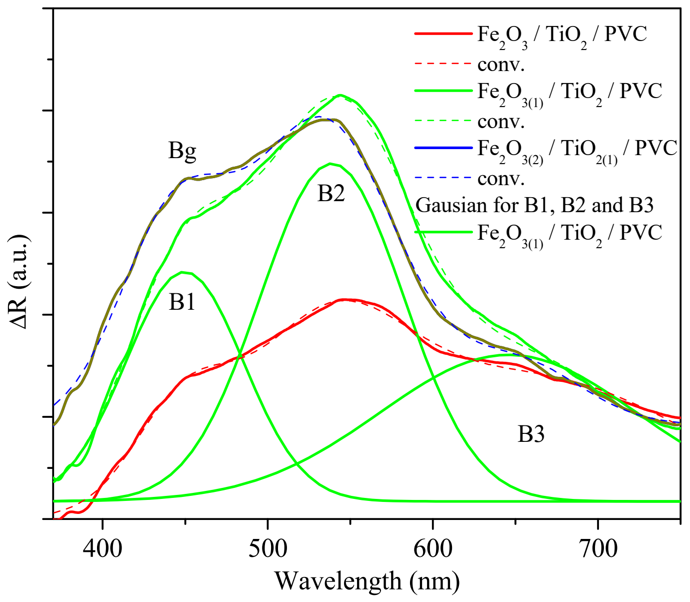

Figure S9. Absorption of $Fe_2O_3/TiO_2/PVC$ (solid lines-measured values, dashed lines-calculated convolutions, B1-B3 deconvoluated bands for $Fe_2O_{3(1)}/TiO_2/PVC$ sample).

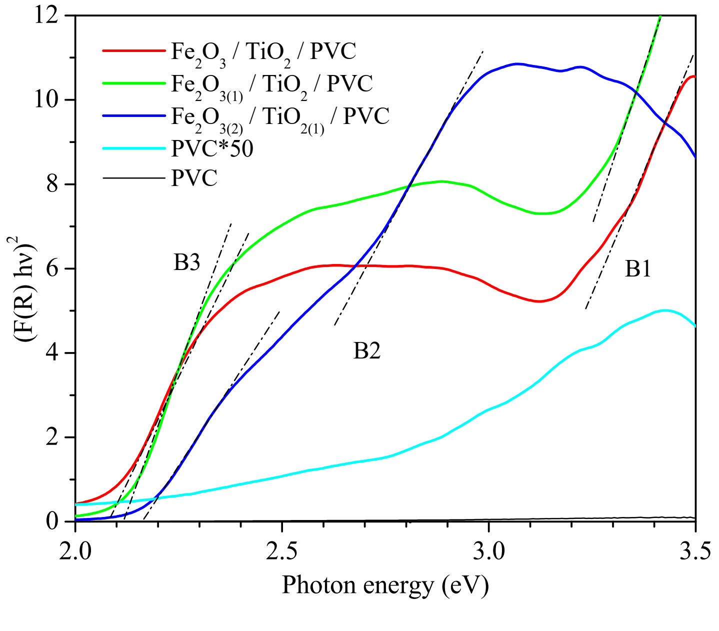

Figure S10. Energy plot of $Fe_2O_3/TiO_2/PVC$ samples.

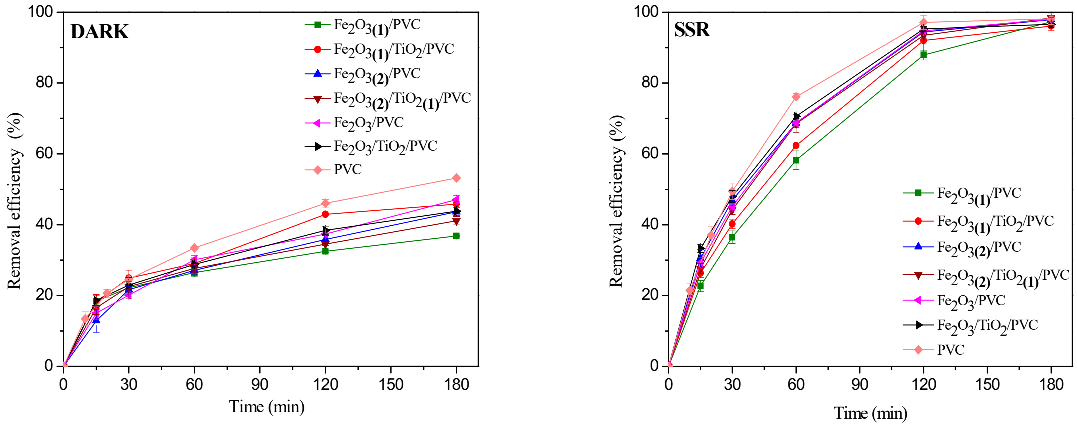

Figure S11. Adsorption and photodegradation kinetic curves for MB removal efficiency (c_0 = 2.45·10^{-2} mM) in the presence of 29 tablets of Fe_2O_3/TiO_2/PVC nanocomposites at a stirring rate of 490 rpm.

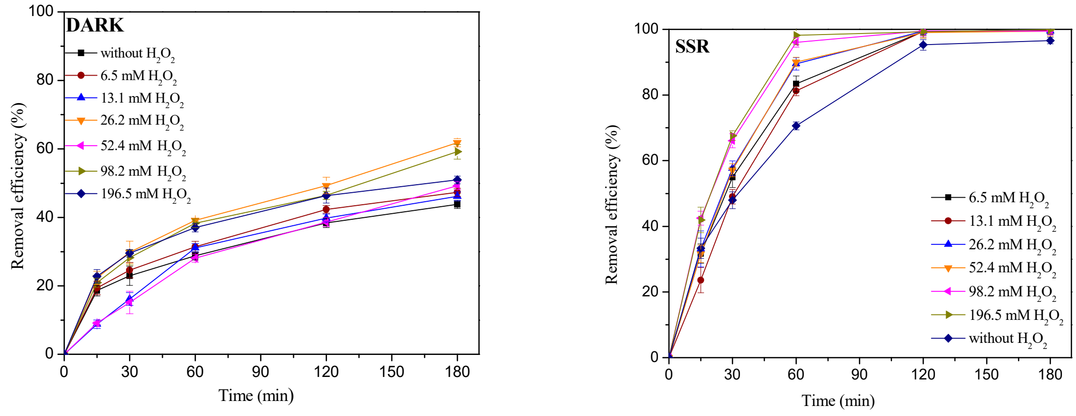

Figure S12. Adsorption and photodegradation kinetic curves for MB removal efficiency (c_0 = 2.45·10^{-2} mM) in the presence of 29 tablets of Fe_2O_3/TiO_2/PVC composites and different H_2O_2 concentrations at a stirring rate of 490 rpm.

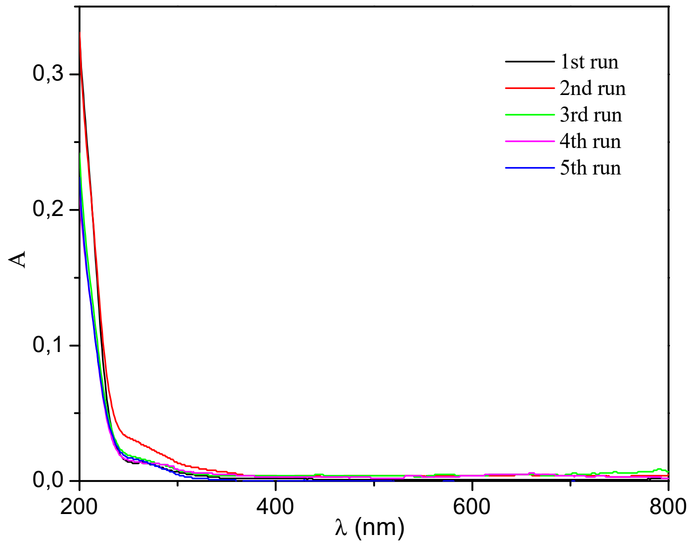

Figure S13. Absorption spectra of ultrapure water left after each photo-cleaning of 29 tablets of Fe_2O_3/TiO_2/PVC composites used in the MB ($c_0 = 2.45 \cdot 10^{-2}$ mM) removal under the influence of UVC irradiation for 60 min.

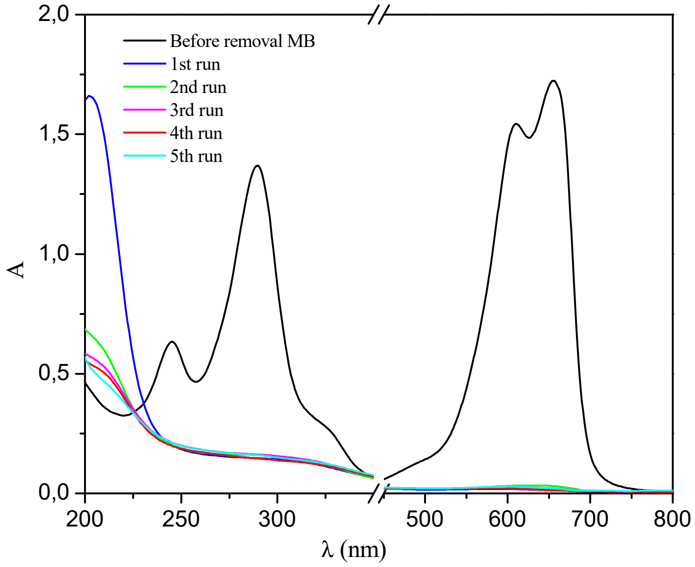

Figure S14. The looks of the absorption spectra of MB ($c_0 = 2.45 \cdot 10^{-2}$ mM) at the beginning and after 180 min of SSR irradiation, at a stirring rate of 490 rpm for five consecutive removals.

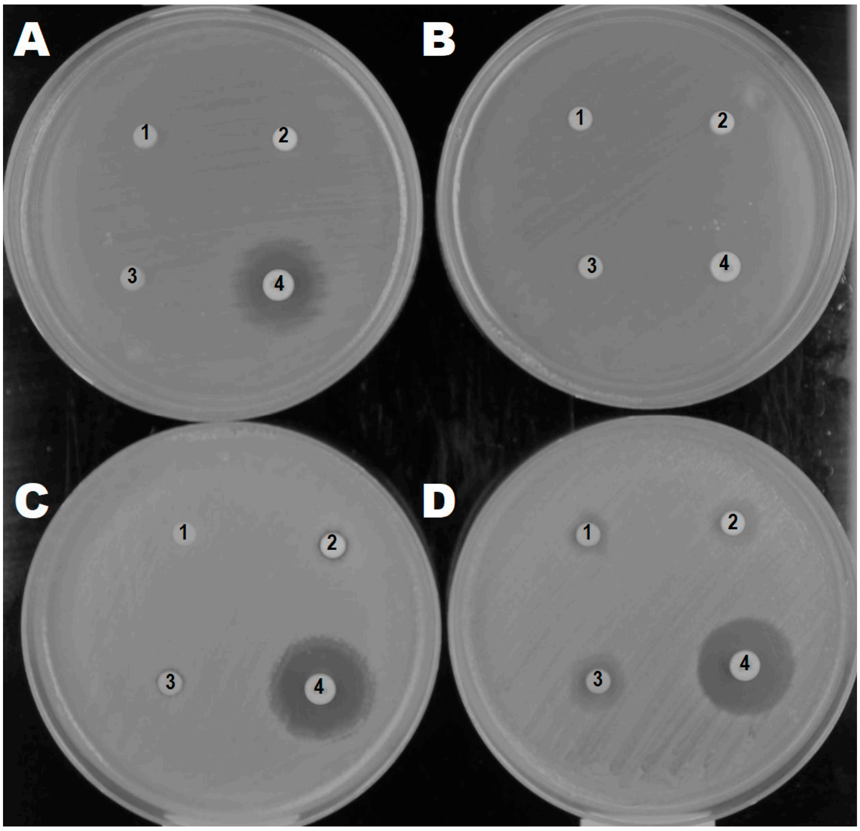

Figure S15. Antibacterial activity of photocatalysts (1 - Fe_2O_3/TiO_2/PVC, 2 – PVC, 3 - Fe_2O_3/PVC, 4 - gentamicin (control)) determined by agar diffusion method: A - *Esherichia coli,* B - *Pseudomonas aeruginosa,* C - *Bacillus cereus,* D - *Staphylococcus aureus*.
